# Mental health service satisfaction among adults with mental illness attending a psychiatric outpatient clinic: a cross-sectional study

**DOI:** 10.3389/fpubh.2025.1471297

**Published:** 2025-01-23

**Authors:** Wondale Getinet Alemu, Lillian Mwanri, Clemence Due, Telake Azale, Anna Ziersch

**Affiliations:** ^1^Flinders Health and Medical Research Institute, College of Medicine and Public Health, Flinders University Adelaide, Adelaide, SA, Australia; ^2^Department of Psychiatry, College of Medicine and Health Sciences, University of Gondar, Gondar, Ethiopia; ^3^Research Centre for Public Health, Equity, and Human Flourishing, Torrens University Australia, Adelaide, SA, Australia; ^4^School of Psychology, The University of Adelaide, Adelaide, SA, Australia; ^5^Institute of Public Health, College of Medicine, and Health Sciences, University of Gondar, Gondar, Ethiopia

**Keywords:** service satisfaction, mental health service satisfaction, factors, outpatient, Ethiopia

## Abstract

**Background:**

Patient satisfaction with services is both a direct and indirect indicator of healthcare quality. It influences healthcare outcomes, patient retention, and the likelihood of medical malpractice claims. However, there is limited evidence on patient satisfaction with mental health services in Africa. Therefore, we aimed to assess mental health service satisfaction and its determinants in adult patients with mental illness in an outpatient clinic in Northwest Ethiopia.

**Methods:**

A hospital-based cross-sectional study was conducted from October to March 2023. Participants were selected using systematic random sampling with a sample interval of three, resulting in a total sample size of 638 invited to participate. Service satisfaction was measured using the interviewer-administered Client Satisfaction Questionnaire-8 (CSQ-8). Data entry, coding, and analysis were performed using SPSS-28. To examine the association of sociodemographic, clinical, social support, and substance use factors, bivariate and multivariate logistic regression analyses were applied. Statistical significance was declared at a *p*-value of <0.05 and 95% CI.

**Results:**

The rate of low mental health service satisfaction among people with mental illness in this study was 24.7%. According to our multivariate logistic regression analysis, people with urban residence 1.77 (1.15, 2.72), poor self-reported health 3.62(1.97, 6.67), having episodic illness ≥2/yr. 0.48 (0.32, 0.74), having relapse 1.75 (1.12, 2.73), and poor drug adherence 2.28 (1.20, 4.35) were more likely to have low mental health service satisfaction than their counterparts.

**Conclusions and recommendation:**

One-quarter of patients with mental illness in the outpatient clinic reported low satisfaction with mental health services. Factors associated with lower satisfaction included urban residency, episodic illness, relapse, poor self-reported health, and poor drug adherence. To enhance patient satisfaction, the clinic should prioritize targeted support for patients facing these challenges.

## Introduction

One of the goals of providing mental health services is patient satisfaction, which is also a key indicator of how well the mental health care system performs (1–4). Patient satisfaction is the degree to which patients feel that their overall medical and healthcare demands are being met (5). Satisfaction is affected by expectations and previous consumer perceptions of the consumption experience (6) and is determined by patient experiences (7). In mental health care, service satisfaction is frequently viewed as a vital process and quality indicator of service (8–11). Overall, evidence suggests that satisfied service consumers adhere to their therapy more rigorously, enabling them to gain more from their care service (12, 13). Conversely, dissatisfied people experience worse treatment outcomes, including nonadherence to therapy (14–16).

Rates of service satisfaction differ between nations. For example, in Europe, a survey conducted in Geneva showed that 93% of patients were satisfied with their outpatient psychiatric care (17). In the Netherlands, patients with psychosis reported low (19.4%), intermediate (48.9%), and high (31.7%) (18) service satisfaction; however, in Denmark, non-Western migrants attending specialized outpatient mental health clinics reported an overall satisfaction of 49% (19). In India, 87% of patients were satisfied with services (20), and in Kuwait, the satisfaction score was 27% in patients with schizophrenia (21). In Israel, 17% of psychotic patients reported being dissatisfied, barely satisfied (46%), moderately satisfied (26%) or delighted (12%) (22) with mental health services. In the African context, satisfaction scores range from 55 to 83% (23), with Ethiopia having satisfaction scores ranging from 57 to 99% (24–26).

Patient satisfaction studies are valuable and crucial means to identify gaps and create practical solutions for improving healthcare services (13). As patient satisfaction increases, trust between the patient and the medical staff also grows (27, 28). Quality of life has also been linked to patient satisfaction. For example, a study conducted in Norway showed that an unmet need for therapeutic relationships and quality of life had a negative association (29), and a review of analyses on quality of life and service satisfaction among psychotic patients indicated that there was a strong association (30) between these two indicators. Another study in Nigeria also showed that service satisfaction was correlated with quality of life (31). Satisfaction also affects treatment adherence, contributing to treatment failure and relapse (32, 33).

Available research on sociodemographic and clinical factors associated with patient service satisfaction comes from North American, European, and Pacific countries (12, 34, 35). There is inconsistent information about the influence of demographic factors, with age (13, 36), sex (13, 37), and education level (13, 37) being associated with satisfaction scores. Other studies have shown no association between these factors and satisfaction with age (19, 31) or sex (19, 31). Waiting time has been associated with satisfaction in some studies (13, 37, 38) but not in others (20). Clinical factors, including severe symptoms (39), a diagnosis of psychosis (37) and a diagnosis of schizophrenia (40), were found to be negatively associated with service satisfaction. Service satisfaction in patients with co-occurrence of mental illness and substance use has been studied in different countries. In the U.S., for example, the satisfaction score for adult patients diagnosed with comorbid mental health problems and alcohol and drug disorders was 29% (41). In Ethiopia, the satisfaction scores of mental illness patients with alcohol use disorder and nicotine dependence were 14.3 and 23.3%, (42) respectively. In a Tanzanian study, patient service satisfaction among patients with mental illness was 32.7% for those also with substance use, 21.7% for those with alcohol use, and 19.8% for those with tobacco and cannabis use disorders (12.9%) (43). These studies indicate that substance use affects service satisfaction for patients with mental illness (44).

Although quantitative research studies have shown that patients have high levels of service dissatisfaction, qualitative studies have shown further insight into patient satisfaction. For example, in Sweden, one study found high levels of service dissatisfaction among mental health service users (45), with evidence demonstrating increased satisfaction among service users who feel supported by staff (46). A study from Europe (Netherlands, Denmark, London, Spain and Italy) showed that service satisfaction was lower for individuals who had less support from family members (47). These studies suggest that it is important to look at a broader range of factors that might influence service satisfaction.

Previous studies on patient satisfaction with mental health services in Ethiopia including the study area differ from our study in several key areas. Notably, prior research did not examine the type of mental illness and its relationship to service satisfaction, history of previous admissions, the impact of episodic illness on satisfaction, or the role of family participation in healthcare on patient satisfaction in study area. Additionally, factors such as age of illness onset, duration of illness, and both subjective and objective illness severity in relation to service satisfaction were not addressed from previous study at study facility. These gaps highlight the need for the current study. This study sought to address a gap in evidence from Ethiopia in relation to associated with service satisfaction, as there has been limited research on this topic and only a narrow range of factors. Therefore, this study aimed to measure the magnitude of mental health service satisfaction among patients attending a psychiatry outpatient clinic in university of Gondar hospital Northwest Ethiopia and to examine the clinical, social support, psychoactive substance uses and sociodemographic predictor factors of satisfaction.

## Methods

### Study design and setting

This cross-sectional study was conducted in Ethiopia among people with mental illness at an outpatient clinic in the Amhara National Regional State at the University of Gondar Comprehensive Specialized Hospital Northwest Ethiopia from October 2022 to March 2023, a public sector institution.

### Source and population

All patients who visited the outpatient clinic of the University of Gondar Hospital were considered the source population, and all patients who visited the outpatient clinic of the hospital during the study period were considered the study population. A total of 638 patients with mental illness who were followed up in outpatient clinics were recruited. Those who had received outpatient care for their mental disorders for at least three months were included, a time period after which patients are more likely to be stable with their condition. Patients with acute mental illness who could not communicate because of severe physical or mental illness were excluded. The data were collected by psychiatry Nurses. The university of Gondar comprehensive specialized hospital serves for the catchment area for more than ten million people and service as referral for district region for more than 15 million people in Northwest Ethiopia.

### Sampling procedure

This paper reports on an examination of service satisfaction within a broader study of quality of life. The sample size was calculated using a single population proportion formula considering the following assumptions. We used a 41% prevalence of poor quality of life from a broader study conducted in Ethiopia, a 95% confidence level, and a 4% margin of error (48).

The following formula was used: *n* = (Zα/2)^2^ * P (1-P)/d2, where n is the minimum sample size needed, Z is a standard normal distribution (*Z* = 1.96) with a confidence interval of 95% and α = 0.05, d is the absolute precision or tolerable margin of error (4%), and P = estimated proportion is assumed to be 41% (0.41). Then, *n* = (1.96)^2^ *(0.41) *(0.59)/(0.04)^2^ = 580 and 10% nonresponse rates (580 *10/100) =58, with a 10 % nonresponse rate of 580 + 58 = 638. We used a systematic random sampling technique to obtain a total sample size of 638 patients who were followed up for the treatment of their mental illness from a group of 2,400 patients who were followed up at the University of Gondar Comprehensive Specialized Hospital, with a sample interval of three. Finally, 636 patients who had been followed up for at least three months and were 18 years old and older were included in the study, and two patients did not complete the study after commencement. Patients who had a clinical diagnosis of schizophrenia, depression, bipolar disorder, anxiety, other psychotic disorders, stress and trauma-related disorders, or somatization disorders were eligible for inclusion.

### Data collection

The data were collected using a standardized questionnaire during a face-to-face interview at the outpatient psychiatry clinic. The questionnaires were prepared in English and translated into the local language, Amharic. Five psychiatry nurses and two MSc psychiatry supervisors participated in the data collection. The data collectors and supervisors received two days of training, and a pre-test of two weeks was conducted but these results were not included in the final analysis. Based on the findings from the pre-test, the questionnaires were revised and finalized. The interview was estimated to take 45 min. As part of the consent process, data collectors sought permission to access the person’s health records, which could assist in providing background information of the patient’s specific diagnosis, medications, and previous history of hospital admission. The data collectors were supervised daily by assigned supervisors, and the filled questionnaires were checked daily by the supervisors and principal investigator. Questionnaires were reviewed daily for completeness by data collectors, supervisors, and then by the researcher throughout data collection. Two incomplete surveys were discarded from the final analysis.

### Variables

Mental health service satisfaction was the dependent variable of this study. The independent variables were sociodemographic characteristics (age, sex, marital status, educational status, living condition, job of participant, residence), clinical factors (type of mental illness, age of onset illness, duration of illness, number of episodes of illness per year, history of admission, comorbid illness, type of drug, drug side effects, counseling, duration of treatment, relapse, objective severity of illness, subjective severity of illness, suicidal ideation, family history of mental illness, waiting time in clinic sand self-reported health), social support-related factors (family not participating in patient care, relationship with family) and substance use factors (alcohol use, tobacco use, family history of substance use). Additional variables, such as self-esteem, drug adherence and legal issues, were also included.

### Measures

#### Service satisfaction

The Client Satisfaction Questionnaire-8 (CSQ-8) comprises an eight-item questionnaire for assessing patients’ global satisfaction with services and has good psychometric properties (49). The CSQ-8 measures general satisfaction on eight scale items ranging from 1 (poor) to 4 (excellent), resulting in a total score ranging from 8 to 32. The level of satisfaction is classified as low (8–20), intermediate (21–25), or high (26–32). Psychometric evaluation of the CSQ-8 in the current sample showed high-scale reliability (Cronbach’s alpha = 0.84). The CSQ-8 has been validated in African Egypt and has internal consistency (Cronbach’s *α* = 0.88) (50).

##### Self-esteem

Self-esteem was measured with the single-item self-esteem scale. The single-item self-esteem scale is a measure of overall self-esteem. Participants rate the single item on a 5-point Likert scale ranging from 1 (not very true of me) to 5 (very true of me). Although shorter than the Rosenberg Self-Esteem Scale, the scale exhibits good convergent and comparable predictive validity (51). This item has not been validated in Ethiopia.

##### Medication adherence

The Medication Adherence Scale (MARS-5) assesses patients’ standard treatment adherence through five questions and five response formats (1 = always, 2 = often, 3 = occasionally, 4 = rarely, and 5 = never). Responses are added for a total score ranging from 5 to 25, with higher scores indicating greater adherence. As advised by the developer, we used a cutoff point greater than or equal to 20 (52, 53) to indicate adherence. This scale has been validated in Africa and Nigeria (54).

##### Substance use

Clients who used substances (for nonmedical purposes) such as alcohol, khat, cigarettes or cannabis in the last year before data collection were considered current substance users (55).

##### Severity of illness

The Clinical Global Impression (CGI) scale of subjective and objective measurement was used to assess the severity of the illness. Responses 1–3 indicate mild, responses 4 indicate moderate, and responses 5–7 indicate severe according to the CGI scale (56). This scale has not been validated in Ethiopia/Africa.

### Data processing and analysis

After verifying accuracy and consistency, all the data were checked, cleaned, coded, and entered into SPSS-28, which was subsequently used to analyses the data. The dependent variable was mental health service satisfaction. First, bivariate logistic regression was used to independently evaluate the relationship between each independent variable and service satisfaction. To control for potential confounding effects, variables with *p* values less than or equal to 0.2 were fitted to a multivariable logistic regression model (57). After adjusting for odds ratios with 95% CIs, bivariate analysis was applied to identify relationships between dependent and independent variables and measure the associations of strength. A *p* value of <0.05 and a 95% CI were used to indicate statistical significance.

### Ethical clearance

Ethical approval was obtained from the Flinders University Human Research Ethics Committee and the Institutional Review Board of the University of Gondar. Written informed consent was obtained from the people who participated in the study. Respondents were briefed and made aware of the study goal and told of their unrestricted freedom to withdraw from the study at any time. Additionally, code numbers rather than personal identification were used to ensure confidentiality.

## Results

### Sociodemographic, clinical, substance use, and social support related characteristics of patients with mental illness in an outpatient clinic in Ethiopia, 2023

The study included 636 participants, yielding a 99.7% response rate. The average age was 35.5 years (±11.7). About half of the participants were female (324, 51%), and most (431, 67.8%) were urban residents. Schizophrenia was the most common diagnosis (274, 43%), followed by depression (192, 30.2%), bipolar disorder (50, 7.9%), anxiety disorder (39, 6.1%), other psychotic disorders (68, 10.7%), somatization disorder (7, 1.1%), and stress/trauma-related disorders (6, 0.9%). Antipsychotic medication was used by 301 participants (47.3%), and 135 (21.2%) were taking both antipsychotics and antidepressants. Of the total patients, 199 (31.3%) had been followed for over five years, nearly 589 (92.6%) reported good self-rated health, 412 (64.8%) experienced relapsing illness, 338 (53.1%) had two or more episodes per year, 316 (49.7%) reported low self-esteem, and 87 (13.7%) had poor drug adherence (Table 1).

**Table 1:** Sociodemographic, clinical, substance use, and social support related factors of people with mental illness in an outpatient clinic in Ethiopia, 2023 (*n* = 636).

Variables	Categories	Frequency (*n* = 636)	Percent (%)
Sociodemographic factors			
Age	Mean ± Sd	35.5 ± 11.7	
Sex			
	Male	317	49.8
	Female	319	50.2
Marital status			
	Married	253	39.8
	Not married.	383	60.2
Educational status			
	Literate	451	70.9
	Illiterate	185	29.1
Living condition			
	Living with family	541	85.1
	Living alone	95	14.9
Job of participant			
	Employed	89	14
	Not employed	547	86
Residence			
	Rural	205	32.2
	Urban	431	67.8
Clinical factors			
Type of mental illness			
	Schizophrenia	274	43.1
		192	30.2
	Depressive disorder	50	7.9
		39	6.1
	Bipolar disorder	68	10.7
		6	0.9
	Anxiety disorder	7	1.1
	Other psychotic disorders		
	Stress/trauma-related disorder.		
	Somatization disorder		
Age of onset illness			
	≤ 25 yrs.	287	45.1
	>25 yrs.	349	54.9
Duration of illness	≤5 yrs.	235	36.9
	>5 yrs	401	63.1
Number of episode/yr.			
	No episode	298	46.9
	≥2 Episode/yr.	338	53.1
History of hospital admission			
	No admission	428	67.3
	Admission	208	32.7
Comorbid illness			
	Yes	77	12.1
	No	559	87.9
Type of drug			
	Antipsychotic	301	47.3
	Antidepressant	123	19.3
	Mood stabilizer	16	2.5
	Anxiolytics	28	4.4
	Antipsychotic and Antidepressant	135	21.2
	Antipsychotic and mood stabilizers	33	5.2
Drug side effect			
	Yes	163	25.6
	No	473	74.4
Counseling			
	Yes	128	20.1
	No	508	79.9
Duration of Rx.			
	≤5 yrs.	437	68.7
	>5 yrs.,	199	31.3
	Yes	412	64.8
	No	224	35.2
Objective severity			
	Mild	493	77.5
	Moderate	94	14.8
	Severe	49	7.7
Subjective severity			
	Mild	424	66.7
	Moderate	159	25
	Severe	53	8.3
	Yes	250	39.3
	No	386	60.7
	Yes	115	18.1
	No	521	81.9
	30 min-1 h.	534	84
	2 h.-3 h.	102	16
Self-reported health			
	Good	589	92.6
	Poor	47	7.4
Social support-related factors			
	Excellent	49	7.7
	Very Good	147	23.1
	Good	299	47
	Fair	100	15.7
	Poor	41	6.4
	Yes	536	84.3
	No	100	15.7
Substance use factors			
	Yes	43	6.8
	No	593	93.2
	Yes	141	22.2
	No	495	77.8
	Yes	78	12.3
	No	558	87.7
Family Hx. Subs.			
	Yes	115	18.1
	No	521	81.9
Additional variables			
Self-esteem			
	Low self-esteem	316	49.7
	High self-esteem	320	50.3
Drug adherence			
	Poor adherence	87	13.7
	Good adherence	549	86.3
	Yes	27	4.2
	No	609	95.8

### Service satisfaction among people with mental illness in an outpatient clinic

The mean and standard deviation of the CSQ-8 score were 23.6 (±4.1), demonstrating intermediate satisfaction. A total of 636 participants [157 (24.7%) reported overall low satisfaction, and 479 (75.3%) reported high satisfaction]. Almost half of the participants (52.8%) said that they received good-quality services; over half (52.5%) reported that they received the kind of service they wanted; some participants (42.6%) reported that the program met their needs; 49.5% of participants would recommend the program to a friend who needed similar help; 50.2% of participants were satisfied with the amount of support they had received; 44.3% patients believed the services they received helped them to deal more effectively with their problems; 38.8% had good satisfaction in a general sense with the service they received; and 49.4% had good responses and would come back if they sought help (Table 2).

**Table 2:** Distribution of items of tools to measure service satisfaction in people living with mental illness in Ethiopia, 2023 (*n* = 636).

	Client Satisfaction Questionnaire (CSQ-8) (Range 8–32)	Poor 1	Fair 2	Good 3	Excellent 4
1	How would you rate the Quality of service received?	2.4%	31.4%	52.8%	13.4%
2	Did you get the kind of service that you wanted?	3%	26.4%	52.5%	18.1%
3	To what extent has our program met your needs?	2%	35.1%	42.6%	20.1%
4	If a friend needed similar help, would you recommend our program to them?	2.4%	18.7%	49.5%	29.4%
5	How satisfied are you with the amount of help you have received?	1.7%	26.6%	50.2%	21.5%
6	Have the services you received helped you to deal more effectively with your problems?	4.1%	25.9%	44.3%	25.6%
7	In an overall, general sense, how satisfied are you with the service you have received?	0.5	26.6%	38.8%	34.1%
8	If you were to seek help again, would you come back to our program?	0.6%	12.9%	49.4%	37.1%

### Factors associated with low mental health service satisfaction

We used binary and multivariate logistic regression to identify factors associated with mental health service satisfaction. As planned, variables with *p* values ≤0.2 in the bivariate analysis were fitted into a multivariate logistic regression model to manage the impacts of confounding effects. A total of twenty-three variables had a p value ≤0.2. The sociodemographic factors (marital status, education, job, residence), clinical factors (number of episodes/yr., number of admissions, comorbid illness, duration of treatment, relapse, suicidal ideation, family history of mental illness, waiting time in clinics, self-reported health, objective severity of illness, subjective severity of illness, self-esteem, drug adherence), substance use factors (family history of substance use, tobacco use, khat use), and social support-related factors (relationship with family, family participates in treatment) were identified through analysis.

Multivariate logistic regression revealed that living in urban regions; having poor self-reported health; having a relapse, having episodic illness; and poor drug adherence were significantly associated with low mental health service satisfaction (Table 3).

**Table 3:** Bivariate and multivariate logistic regression analysis of factors associated with low mental health service satisfaction among people with mental illness in Ethiopia, 2023 (*n* = 636).

Variables	Categories	Satisfaction		Crude odd ratio (COR 95%CI)	Adjusted odd ratio (AOR 95%CI)	*P* - value
		High	Low			
Marital status	Married not married	199 280	54 103	1 1.35 (0.93, 1.97)	1 1.29 (0.86, 1.92)	0.112
Living condition	Living with family Living alone	413 66	128 29	1 1.41 (0.87, 2.29)	1 1.01 (0.55, 1.88)	0.152
Education	Literate Illiterate	337 142	114 43	1 0.89 (0.59, 1.33)	1 1.14 (0.67, 1.93)	0.589
Job	Employed Not employed	61 418	28 129	1 0.67 (0.41, 1.09)	1 0.74 (0.43, 1.29)	0.110
Residence	Urban Rural	317 162	114 43	1.35 (0.90, 2.01) 1	1.77 (1.15, 2.72) * 1	0.009
Number of episode/yr.	No episode ≥2 Episode/yr	215 264	83 74	1 0.72 (0.50, 1.04)	1 0.48 (0.32, 0.74) *	0.001
Hospital admission	No admission Admission	320 159	108 49	1 0.91 (0.62, 1.34)	1 0.77 (0.50, 1.19)	0.646
Comorbid illness	No yes	430 49	129 28	1 1.90 (1.15, 3.15)	1 1.50 (0.85, 2.64)	0.111
Duration of Rx.	≤5 yrs. >5 yrs.	332 147	105 52	1 1.11 (0.76, 1.64)	1 0.80 (0.50, 1.27)	0.568
Relapse	No Yes	182 297	42 115	1 1.67 (1.12, 2.50)	1 1.75 (1.12, 2.73) *	0.013
Suicidal ideation	No Yes	301 178	85 72	1 1.43 (0.99, 2.06)	1 1.13 (0.74, 1.71)	0.053
Family Hx. MI	No Yes	376 103	116 41	1 1.29 (0.85, 1.95)	1 1.04 (0.63, 1.72)	0.231
Family Hx. Subs.	No Yes	398 81	123 34	1 1.35 (0.86, 2.12)	1 1.10 (0.63, 1.91)	0.180
waiting time in clinics	30 min-1 h. 2 h.-3 h.	396 83	138 19	1 0.65 (0.38, 1.12)	1 0.70 (0.38, 1.27)	0.121
Rate your health	Good Poor	524 30	65 17	1 3.93 (2.14, 7.20)	1 3.62 (1.97, 6.67) *	0.001
R/ship with family	Excellent Very Good Good Fair Poor	43 123 220 70 23	06 24 79 30 18	1 1.39 (0.53, 3.65) 2.57 (1.05, 6.27) 3.07 (1.18, 7.98) 5.60 (1.95, 16.08)	1 0.97 (0.34, 2.75) 1.21 (0.44, 3.32) 1.37 (0.46, 4.06) 2.16 (0.65, 7.19)	0.301 0.826 0.618 0.142
Family participates patient care	Yes No	410 69	126 31	1 1.46 (0.91, 2.33)	1 0.99 (0.54, 1.82)	0.984
Objective severity	Mild Moderate & Severe	379 100	114 43	1 1.43 (0.94, 2.16)	1 0.96 (0.56, 1.64)	0.896
Subjective severity	Mild Moderate & Severe	331 148	93 64	1 1.53 (1.06, 2.23)	1 1.23 (0.79, 1.90)	0.326
Tobacco use	No Yes	454 25	139 18	1 2.35 (1.24, 4.43)	1 1.86 (0.94, 3.66)	0.320
Khat use	No Yes	375 104	120 37	1 1.52 (0.91, 2.54)	1 0.77 (0.37, 1.58)	0.419
Self-esteem	High self-esteem Low self-esteem	249 230	71 86	1 1.31 (0.91, 1.88)	1 1.09 (0.71, 1.65)	0.689
Drug adherence	Good adherence Poor adherence	406 73	143 14	1 1.83 (1.00, 3.35)	1 2.28 (1.20, 4.35) *	0.012

* Variable which have significant association with dependent variable.

Participants from urban regions were 1.77 times more likely (AOR = 1.77, 95% CI: 1.15–2.72) to report lower satisfaction with services compared to those from rural regions.

Patients who self-reported poor health were 3.62 times more likely (AOR = 3.62, 95% CI: 1.97–6.67) to report low service satisfaction compared to those who reported good health.

The odds of low mental health service satisfaction were 1.75 times higher (AOR = 1.75, 95% CI: 1.12–2.73) among participants with relapsed illness compared to those without relapsing illness.

Patients with non-episodic illness were 48% less likely (AOR = 0.48, 95% CI: 0.32–0.74) to experience low mental health service satisfaction compared to those with episodic illness.

Participants with poor drug adherence had 2.28 times higher odds of low mental health service satisfaction (AOR = 2.28, 95% CI: 1.20–4.35) compared to those with good drug adherence.

## Discussion

Since service satisfaction is one of the factors that can affect service user quality of life and health outcomes, measuring outpatients’ satisfaction with mental health services in psychiatric clinics is essential. Furthermore, by measuring patient satisfaction, institutions may recognize areas were service delivery needs improvement. The current study was designed to determine mental health service satisfaction and associated factors among patients attending a psychiatry outpatient clinic.

The overall magnitude of high mental health service satisfaction was 75.3% (72–79.1%), while the remaining 24.7% reported low service satisfaction. This magnitude is similar to that of a previous study conducted University of Gondar Hospital in 2017, in which approximately 77.6% of the study participants were satisfied with outpatient care (58). These findings were also consistent with those of another study conducted in an outpatient clinic receiving mental health services at public hospitals in Mekelle town, Ethiopia, which reported a satisfaction rate of 72% (37). This similarity may be because of both studies likely shared similar factors influencing patient satisfaction, such as the quality of care, the approach of patients and healthcare providers, and access to medications and service availability. Additionally, the facilities in both studies were specialized hospitals with similar characteristics, which may have influenced patient satisfaction levels. The similarity of results suggests that common factors, such as improvements in service delivery and the availability of patient support, are key contributors to satisfaction in outpatient mental health services.

The satisfaction score was higher in our study than in other psychiatric outpatient hospital settings in Ethiopia, for example, at St. Poul Hospital in Ethiopia (50.3%) (59), university of Gondar Hospital (65.46%), at Dilla Hospital (55.4%) (38) and at Dessie Hospital (61.2%) (60). The discrepancy in satisfaction scores might be attributable to factors such as the use of different measurement tools, differences in mental health literacy among patients and mental health services, and differences in actual service quality of health care facilities. In general, mental health service satisfaction varies across Ethiopia due to significant differences in health infrastructure, the availability of mental health professionals, and access to essential medications. Urban areas have better facilities and more specialized services compared to rural regions. Additionally, cultural beliefs about mental illness differ across communities, influencing how people perceive and utilize mental health services. In some areas, higher levels of stigma toward mental illness cause to lower satisfaction, as people may face reduced social support and understanding (61, 62). The higher satisfaction score in our study, compared to other psychiatric outpatient settings in Ethiopia, could be attributed to several factors. Our study site has seen recent improvements in service delivery, including better patient-staff communication. As referral hospital, it benefits from senior professionals providing accessible care like psychiatrist, mental health professionals, clinical psychologist and social workers and psychiatry Nurses. additionally, the availability of medications and other resources, such as support services, plays a key role in patient satisfaction. Difference in similar setting may be related to time difference in the study, prior studies done before five years ago due to that there is quality of care difference in the service provided that cause difference in mental health service satisfactions.

When asked about their satisfaction specifically with the quality of care that was provided, almost half of the survey participants (52.8%) reported receiving good quality services in mental health care in the study setting. This observation was similar to the findings of a hospital-based study in Ethiopia, where almost 50% of patients were satisfied with the quality of service received in a psychiatry outpatient clinic, and better than the findings of another study conducted in Ethiopia on the perceived quality of a general medical outpatient service, where only 35% of participants reported receiving good quality service (63). These differences could have been a result of a variation of care in different settings, i.e., between services provided in specialized psychiatry outpatient clinics and those provided by general medical outpatient services, with specialized clinics receiving higher satisfaction scores compared to general medical services where electroconvulsive therapy and senior specialists were present.

According to international comparisons, the service satisfaction score in our study was greater than that reported in studies conducted among patients with mental illness in outpatient clinics in other African countries, including 50% in Egypt and 52.4% in Nigeria (64). Differences in service satisfaction scores across these studies may come from variations in data collection methods and study designs. For instance, while other studies included all patients attending psychiatric outpatient clinics, the Nigerian study focused exclusively on patients with schizophrenia. This narrower cohort of participants may have influenced the satisfaction scores.

Other studies across different continents have reported varying satisfaction scores; for example, in a study conducted in Pakistan, 72% of participants were satisfied with mental health services and in a study conducted in Geneva, 85% of patients reported satisfaction with their mental health outpatient treatment (17). Furthermore, equivocal findings have been demonstrated in different studies that aimed to measure mental health service satisfaction in more developed settings globally; for example, 45% of anxiety outpatients in Los Angeles, 53% of patients with mental health in Mexico and 52% of patients with mental illness in Ireland (65). These differences might be attributable to factors such as the study setting, cultural context and patient expectations of the facilities (36); differences in the data collection methods and study design; and variation in the satisfaction measurement tools used.

In terms of the factors associated with service satisfaction, the satisfaction level of participants in this study was significantly related to area of residence. Participants who were from urban areas were 1.77 times more likely to report low mental health service satisfaction than were those who came from rural areas. Previous studies have reported similar findings in mental health care services in Ethiopia; urban residence was found to be a factor associated with low satisfaction with mental health services provided in an outpatient psychiatry service of Dilla University referral hospital in southern Ethiopia (38), and in Dessie, those living in urban areas were less likely to be satisfied (60). This may be related to urban residents often have higher expectations for service quality due to the greater availability of resources and better infrastructure. Although urban areas offer a wide range of mental health services, in my opinion rural areas may benefit from stronger social networks and family or community-centered support systems. In addition, in my opinion informal support systems in rural areas are common and it can supplement formal care service, leading to higher patient satisfaction and future research should focus to study between service satisfaction and residency status.

This study found that patients with relapsed illness were 1.75 times more likely to report low satisfaction with mental health services compared to those without a relapsing illness. To our knowledge, no previous quantitative studies have directly compared these outcomes with our findings. It may be that patients who experience relapse may feel that the treatments and support provided are not effectively meeting their needs, leading to frustration and disappointment with mental health services. In addition, the increased time and financial demands associated with relapse can add stress, further affecting their overall satisfaction. Additionally, relapse may intensify feelings of stigma, which can lower their satisfaction with the care received (66, 67).

In this study, patients with poor self-rated health were 3.62 times more likely to report low satisfaction with mental health services compared to those with good self-rated health. This could be attributed to individuals with mental illness and poor self-rated health often experiencing life dissatisfaction, low mood or depression, memory and concentration difficulties, and challenges in maintaining positive social relationships with family, friends, and neighbors (68). These findings align with a national survey conducted in Australia, which explored poor self-rated health and its association with somatization disorder (69).

The results obtained from this study showed that patients who had poor drug adherence were more than twice as likely to have low mental health service satisfaction than participants with good drug adherence. Previous studies have reported similar findings showing that drug nonadherence is associated with service dissatisfaction, an indication that patients with mental illnesses may be more prone to not taking their medications as prescribed and may consequently develop low service satisfaction (70).

Studies considering factors associated with mental health service satisfaction have previously shown a statistical correlation between the short waiting time for services in the hospital (71–75). In this study, approximately 157 patients (25%) had low satisfaction with the wait time at the outpatient clinic; however, waiting time was not significantly associated with patient satisfaction in the final analysis. In contrast, another study in Ethiopia that was conducted in a medical outpatient clinic showed that having a waiting time of less than one hour was a predictor of perceived good quality care by patients with mental illness (63).

Notably, in the present study, unlike in other studies, age, marital status, education, job, type of diagnosis, suicidal ideation, and family history of mental illness were not significantly correlated with low service satisfaction among participants. Previous Ethiopian studies have shown the following variables to be associated with service satisfaction: being male, being widowed, having been diagnosed with schizophrenia, having poor social functioning, living a long distance from the hospital, waiting for the service for a long time, having a low monthly income (38), not being hospitalized, receiving medications from the hospital and having strong social support (76). The cause may be attributed to variations in the diagnosis of patients receiving care at facilities, and potential methodological variations in the study method should also be considered.

### Strengths and limitations of the study

This study has several strengths and limitations. One of the strengths of this study is that this is the first study to show overall mental health service satisfaction among patients with mental illness in an outpatient clinic of a comprehensive specialized hospital in Ethiopia, including patients with different clinical diagnoses, such as schizophrenia, depression, bipolar disorder and other psychosis disorders. This is the first study to comprehensively examine the impact of sociodemographic, clinical, substance use, and social support-related factors on patients’ satisfaction with mental health services in Ethiopia. Additionally, factors such as relapse, episodic illness, and self-rated health have shown an association with low mental health service satisfaction. These are new factors that had not been previously assessed as predictors of low mental health service satisfaction among patients with mental illness.

This study has several limitations that should be considered, including (i) social desirability bias that could affect the questionnaire responses and data collectors are clinical stuffs working in the study area (ii) its cross-sectional nature and ability to determine the temporal relationship between patient satisfaction and related parameters, and (iii) the ability of the clinic to recruit patients who were more likely to be satisfied, the effect of which could have increased the satisfaction score for the study and lastly the study excluded severely ill patient who did not communicate that makes the result biased. It is also possible that those patients who were not satisfied with the service provision may have not attended the follow-up in the outpatient clinic and were therefore missing among the study participants.

### Conclusion and recommendations

The findings of this study showed that almost 25% of patients in outpatient clinics had low mental health service satisfaction, which requires improvement. Those from urban residences with episodic illness; with a relapse illness; with self-reported health, poor and good health; and poor drug adherence were found to have lower mental health service satisfaction. Therefore, hospital administrations, lawmakers, and accountable authorities should work on activities that increase patient satisfaction, considering these factors. Future research should focus on to study the relationship between service satisfaction and some variables like residence being urban and rural, and informal and formal support.

## Data Availability

The raw data supporting the conclusions of this article will be made available by the authors, without undue reservation.
